# T4/N2 classification nasopharyngeal carcinoma benefit from concurrent chemotherapy in the era of intensity-modulated radiotherapy

**DOI:** 10.18632/oncotarget.11981

**Published:** 2016-09-12

**Authors:** Ruifei Xie, Bing Xia, Xuebang Zhang, Wei Hu, Ruping Zhao, Congying Xie, Jianhua Wang, Ni Zhang, Shixiu Wu

**Affiliations:** ^1^ Hangzhou Cancer Hospital, Hangzhou, Zhejiang, P. R. China; ^2^ Department of Radiation Oncology, Wenzhou Medical College Cancer Center, Wenzhou, Zhejiang, P. R. China; ^3^ Department of Radiation Oncology, Taizhou Central Hospital, Zhejiang, P. R. China

**Keywords:** nasopharyngeal carcinoma, propensity score matching, intensity modulated radiotherapy, concurrent chemotherapy

## Abstract

Although the benefits of concurrent chemotherapy (CC) in the treatment of locally advanced nasopharyngeal carcinoma (NPC) had been proven in the era of two-dimensional radiotherapy, long-term efficacy and safety of using CC combined with intensity-modulated radiotherapy (IMRT) remain unclear. A retrospective analysis of 1,182 patients who underwent IMRT for clinical II-Iva NPC was performed. Propensity score matching algorithm was used to identify two matched cohorts with or without CC (264 patients per cohort). Median follow-up time was 45.6 and 43.6 months for the two cohorts. The estimated 5-year overall survival rate was 81.8% (95% CI 76.6-87.4) in patients treated with CC and 73.7% (95% CI 67.8-80.0) in those treated without CC, respectively (hazard ratio 0.64, 95% CI 0.44–0.93; *p* = 0.018). The benefit of CC was mainly observed in those patients with good performance status, male, age > 48 years, T4 and N2 classification. Grade 3/4 acute toxicities were more common in those patients administrated with CC. The grade and incidence of late salivary glands damage were also increased by CC (*p* = 0.003). These findings indicated that the addition of CC significantly improved treatment outcomes of NPC patients treated with IMRT, but accompanied increased toxicities. Tailored CC and optimizing schedule of IMRT and systemic therapy were needed, provided that distant metastasis was the predominant pattern of failure in patients treated with IMRT.

## INTRODUCTION

Nasopharyngeal carcinoma (NPC) is relatively uncommon worldwide, but endemic in Southeast Asia, the Arctic, and the Middle East/North Africa [1]. In 2015, it was estimated that 60,600 people would be diagnosed with NPC and 34,100 patients would die because of the disease in China, which constitutes a major public health burden [2]. Radiotherapy is the primary and only curative treatment modality in the management of NPC because of its radiosensitivity and anatomical specificity. Over the past three decades, the prognosis of NPC has dramatically improved because of advances on imaging and radiotherapy techniques, and the wide application of systemic chemotherapy [1, 3].

The introduction of intensity-modulated radiotherapy (IMRT) in the treatment of NPC has generated much more excitement with higher local-regional control and less radiation-related sequel, due to improved coverage of tumor target and spare of normal structures. With IMRT, local-regional control rates exceeding 90% has been documented and the overall survival rate was more than 80% in recent studies [4-7]. Currently, for early stage disease (I & IIa stage), IMRT alone achieved satisfied local control and a good survival outcome; for advanced stage disease (III & IVa stage), concurrent chemotherapy (CC) combined with IMRT is often recommended empirically in clinical practice [1, 3, 4].

However, the strategy of using concurrent chemoradiotherapy was established as the standard of care for locally advanced NPC in the two/three dimensional radiation techniques. Question remains whether IMRT combined with CC improves the therapeutic effect over and above IMRT alone. Even more concerning, incremental acute and late toxic effects were reported when CC was delivered with IMRT. The aim of this study is to investigate the role of CC in NPC patients treated with IMRT.

## PATIENTS AND METHODS

### Patients

We performed a retrospective analysis of 1,182 patients who underwent IMRT with or without CC for clinical II-Iva NPC from 2002 to 2009 in three cancer centers in Zhejiang Province of China (First Affiliated Hospital of Wenzhou Medical College, Hangzhou Cancer Hospital and Taizhou Central Hospital). Approval for this study was provided by the institutional review board of three hospitals, and individual patient consent was waived. All patients had complete pretreatment evaluation including clinical history, physical examination, biochemical test, fiberoptic nasopharyngoscopy with biopsy, magnetic resonance imaging (MRI) or computer tomography (CT) scan of the head and neck, chest radiography, and ultrasonography of the abdominal region. Additional investigations were performed if indicated. Stage data was retrieved from the original chart reports and was unified to the seventh edition of the International Union against Cancer/American Joint Committee on Cancer (UICC/AJCC) staging system for NPC; in case of incomplete stage information, original images were retrieved and reviewed by our study radiation oncologists.

### Treatment

The techniques of planning and delivery of IMRT used in the center were similar and described previously [8]. Briefly, all patients were immobilized with specified device; contrast-enhanced planning CT scans with a 3-mm slide thickness were then obtained. All target volumes were outlined slice by slice on the axial contrast-enhanced CT images in the treatment planning system. Diagnostic MRI images were coregistered with the simulated CT images to assistant target delineation. The target volumes were defined in accordance with the International Commission on Radiation Units and Measurements Reports 50 and 62.

Simultaneous modulated accelerated radiotherapy technique was adopted. The prescribed dose was 68-70 Gy/28-30 fractions to the gross target volume, 56-60 Gy/28-30 fractions to the high-risk clinical target volume, and 45-54 Gy/23-30 fractions to the low-risk clinical target volume. The range of radiation dose per fraction for the gross target volume was 2.3-2.5 Gy, and the high daily dose of 2.5 Gy was based on our previous study [8].

Although the benefits of CC in the treatment of locally advanced NPC had been proven in the era of two-dimensional radiotherapy, the use of CC with IMRT was not widely accepted in this region, provided that good local-regional control with IMRT alone and high toxicity incidence rates when CC was delivered. With more and more evidence supported using CC for advanced NPC and accumulation of experience in the management of toxicities related to concurrent chemoradiotherapy, this strategy was accepted gradually in clinical practice but often with a reduced dose of chemotherapy. Generally, CC was delivered with cisplatin alone (80 mg/m^2^/d on days 1 and 22 or 25 mg/m^2^/d weekly). Adjuvant chemotherapy consisted of gemcitabine plus cisplatin, or paclitaxel/docetaxel plus cisplatin given every three weeks for two to three cycles.

### Follow-up

Follow-up evaluations occurred every 3 months during the first 2 years; every 6 months during years 3 to 5; then annually. Routine evaluations included a complete physical examination, nasopharyngoscopy or an indirect nasopharyngeal speculum examination, a biochemical profile, annual chest radiography, abdominal ultrasonography, and a head-and-neck CT/MRI. Systemic/acute radiation adverse effects were scored by using the National Cancer Institute Common Toxicity Criteria (CTCAE, version 4.0) [9], whereas late radiation effects were evaluated according to Radiation Therapy Oncology Group/European Organisation for Research and Treatment of Cancer (RTOG/EORTC) criteria late radiation morbidity scoring schema [10].

### Statistical analysis

To address the imbalance of potential confounders between groups with and without CC, propensity score matching was used to create 2 treatment cohorts with balanced distribution of baseline characteristics [11]. The variables for the propensity score matching were choosing by first performing a logistic regression of using CC versus without CC on the following factors: age (years), sex (male vs female), ECOG performance status (0-1 vs 2), adjuvant chemotherapy (yes vs no), gross tumor volume, T-stage, N-stage, data of IMRT (2002-2005 vs 2006-2009). A revised Charlson comorbidity index, named as head and neck comorbidity index score (HN-CCI, 0 vs 1-2 vs 3-6), was also included in order to control for comorbidity [12]. The potential predictors that were not statistically significant (p > 0.05) were removed, and propensity scores were calculated from the logistic regression refit to the reduced variable group. Patients administrated with and without CC were then matched using a one-to-one nearest neighbour calliper of width 0.025 (maximum allowable difference in propensity scores). Only patients matched with propensity scores were included in the subsequent analyses.

Covariates balances between the two sets were examined by t test (continuous variable), X^2^ test or Fisher's exact test (categorical variable) as appropriate. Survival rates were calculated using the Kaplan-Meier method and the log-rank test was used for comparison in the two sets. Multivariate analyses with the Cox proportional hazards model were used to calculate hazard ratios (HR), 95 % confidence intervals (CI). Two-tailed *p*-values < 0.05 were considered statistically significant.

## RESULTS

### Patients

Among the 1182 patients treatment with IMRT, 576 (48.7%) and 606 (51.3%) patients were administrated with and without CC, respectively. The significant potential factors of choosing CC included young age, male, advanced T and N stage and no adjuvant chemotherapy. After propensity score matching, 264 (50%) patients treated with CC and 264 (50%) patients treated without CC remained in the two cohorts. The included patients had highly balanced characteristics, as showed in Table 1. All subsequent analyses were based on the propensity-matched cohorts.

**Table 1:** Baseline characteristics of nasopharyngeal carcinoma patients underwent IMRT with or without concurrent chemotherapy

	Before propensity score matching				After propensity score matching		
Subgroup		Without CC	With CC	*P* value	Without CC	With CC	*P* value
Number		606	576		264	264	
Stage	II	192	50	<0.001	43	36	0.694
	III	270	320		145	149	
	IV	144	206		76	79	
T stage	1	36	42	<0.001	12	12	1
	2	267	122		77	77	
	3	182	247		108	108	
	4	121	165		67	67	
N stage	0	167	78	<0.001	46	52	0.622
	1	225	195		104	105	
	2	172	257		93	93	
	3	42	46		21	14	
Sex	Male	421	446	0.002	199	191	0.489
	Female	185	130		65	73	
ECOG PS	0-1	280	281	0.407	121	139	0.139
	2	326	295		143	125	
Adjuvant chemotherapy	No	502	357	<0.001	200	183	0.119
	Yes	104	219		64	81	
GTV (cm^3^)	Median	27.6	35.6	<0.001	31.3	31.9	0.8701
	range	0.9-182.7	1.1-250.5		1.8-150.2	3.2-156.0	
Age (years)	median	50	47	<0.001	48	48	0.741
	range	13-83	13-75		13-74	26-75	
HN-CCI	0	290	257	0.176	121	120	0.596
	1-2	214	198		82	91	
	3	102	121		61	53	
Date for IMRT (year)	2002-2005	354	342	0.783	144	158	0.218
	2006-2009	252	234		120	106	

Abbreviation: CC, concurrent chemotherapy; ECOG PS, performance status; GTV, gross tumor volume; HN-CCI, head and neck comorbidity index score; IMRT, intensity-modulated radiotherapy.

### Survival outcomes

**Figure 1:**
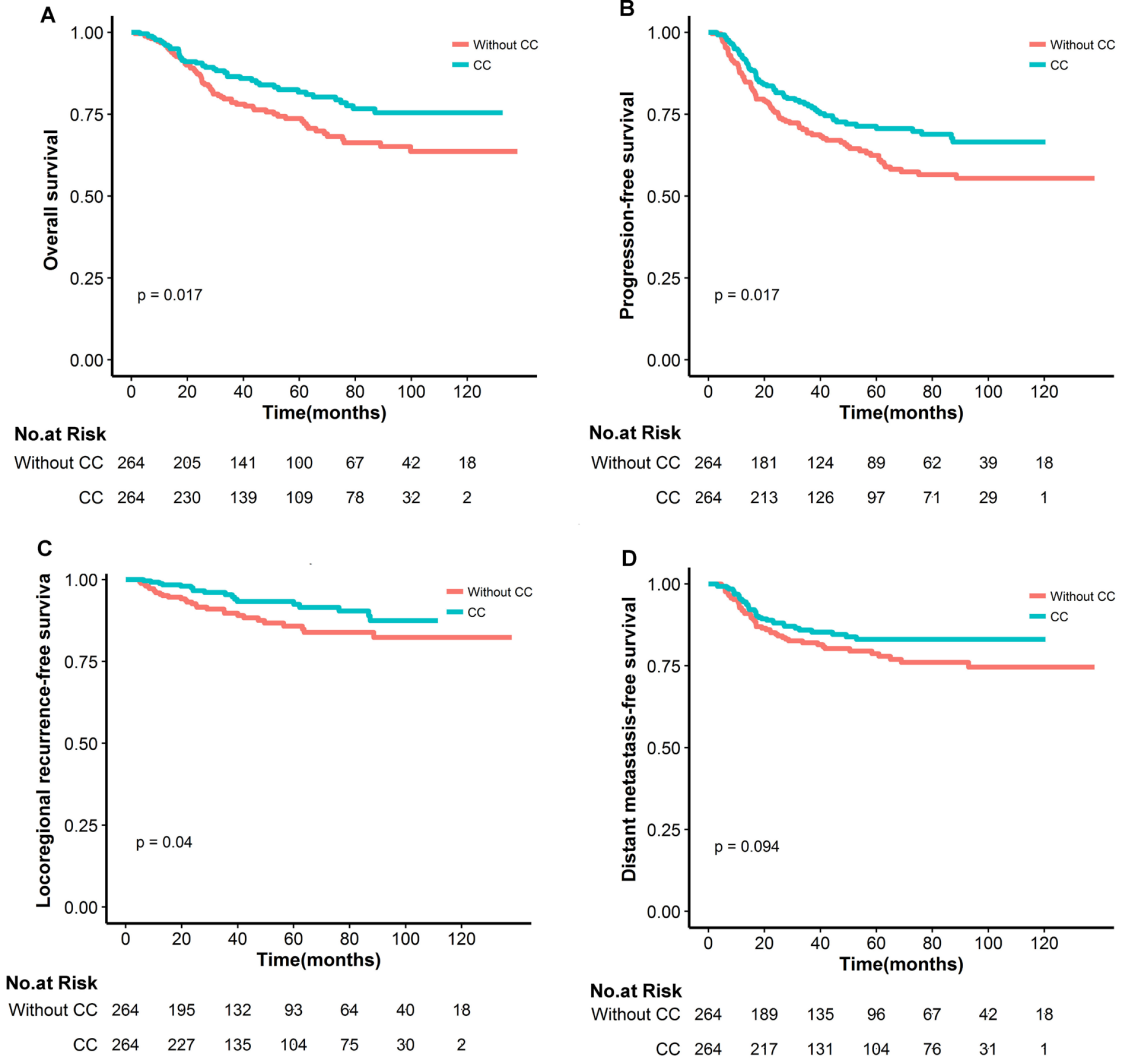
Comparison of overall (A), progression-free (B), locoregional recurrence-free (C) and distant metastasis-free survival curves in nasopharyngeal carcinoma patients underwent IMRT with or without concurrent chemotherapy(CC)(D)

The median follow-up time was 45.6 months (range 3.0-132.7 months) and 43.6 months (range 3.7-138.0 months) for the patients treated with and without CC, respectively. The estimated 5-year overall survival rates were 81.8% (95% CI 76.6-87.4) in patients treated with CC and 73.7% (95% CI 67.8-80.0) in those treated without CC, respectively (HR 0·64, 95% CI 0·44-0.93; *p* = 0·018). The patients administrated with CC had significantly improved progression-free and locoregional failure-free survival compared with those without CC (both *p* < 0.05). Although a favorable trend toward reduced distant recurrence in those patients treated with CC, the difference did not reach a statistical significance (*p* = 0.094) (Figure 1).

**Figure 2:**
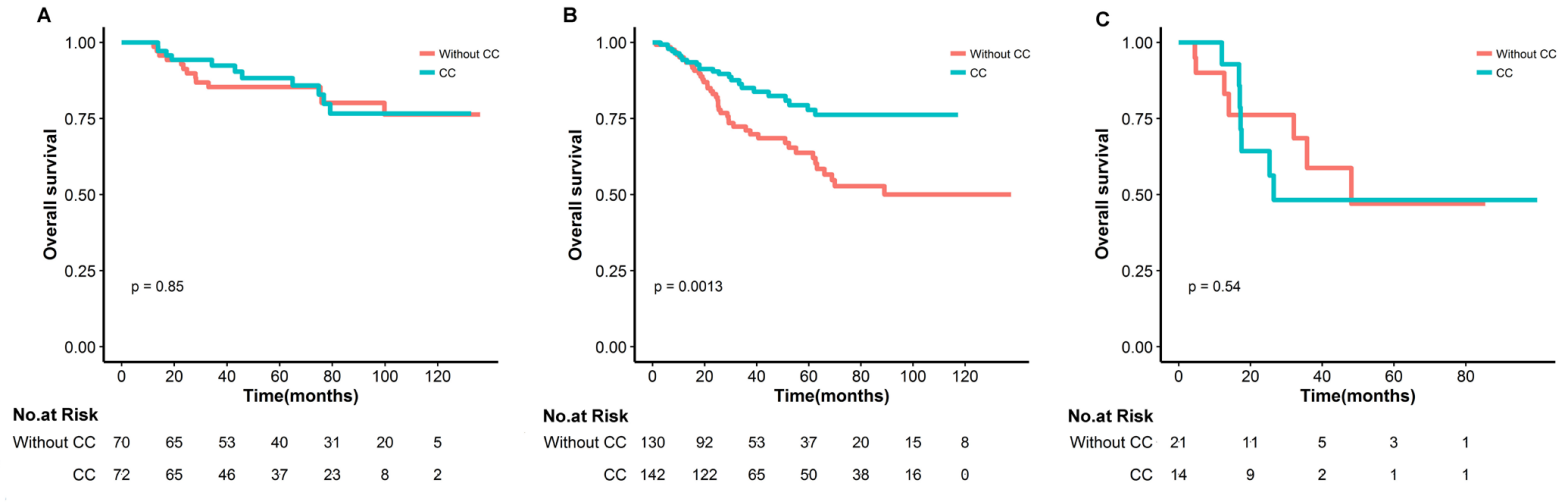
Kaplan-Meier survival curves in nasopharyngeal carcinoma subgroups underwent IMRT with or without concurrent chemotherapy for T3N0-1(A), T4N0-1/T1-4N2(B) and T1-4N3(D)

In the subsequent subgroup analysis, over survival was chosen as the endpoint to explore the potential population who might benefit from IMRT and CC, considered that the nature and limitation of tumor-centered clinical endpoints, like tumor control or distance metastasis. The advantage of CC was mainly observed in those patients with good performance status, male, age > 48 years, T4 and N2 classification (Figure 2). There was no clear evidence that any subgroup of patients defined by HN-CCI, date of IMRT, adjuvant chemotherapy and gross tumor volume benefited more or less from CC in terms of overall survival. The estimated 5-year overall survival rates for patients with T4N0-1 and T1-4N2 disease were 77.8% in the group treated with CC and 63.8% in those treated without CC, respectively (HR 0.50, 95% CI 0.27-0.74, *p* < 0·002). There was no difference in overall survival between the two cohorts for other subgroups (T3N0-1 or T1-4N3 stage, Table 2).

**Table 2:** The estimated 5-year overall survival rates in subgroups underwent IMRT with or without concurrent chemotherapy

Subgroup	Without CC(No^a^)	With CC(No^a^)	HR (95CI%)	*P* value
T3N0-1	85.3% (70)	88.3% (72)	0.92(0.41-2.07)	0.847
T4N0-1/T1-4N2	63.8% (130)	77.8% (142)	0.45(0.27-0.74)	0.002
T1-4N3	47.0% (21)	48.2% (14)	1.41(0.48-4.16)	0.537

Abbreviations: IMRT, intensity-modulated radiotherapy; CC, concurrent chemotherapy; CI = confidence interval.

a The number of patients in subgroups.

### Pattern of first failure

At their last follow-up visit, a total of 131 (24.8%) patients experienced disease progression, the majority of which was distant metastasis with or without locoregional recurrences (15.9%). In the patients treated with CC, 17 (6.4%) and 38 (14.4%) patients had developed locoregional failures and distant metastases, respectively. In the patients treated without CC, 30 (11.4%) and 46 (17.4%) patients had developed locoregional failures and distant metastases, respectively.

### Toxicity

There were no treatment-related deaths. Acute toxicities were assessed in all of the patients (Table 3). Overall, CC using cisplatin during IMRT was well tolerated; the total dose of the patients with CC included in this analysis ranged from 125mg/m^2^ to 160mg/m^2^ and no one received 1 cycle of CC. Notably, grade 3/4 of leukopenia, thrombocytopenia, mucositis and nausea/vomiting was more common in patients delivered IMRT with CC, compared with those without CC.

At 2 years after treatment, late toxicities were available in 128 patients with CC and 116 patients without. The grade and incidence of late salivary glands toxicities were increased by CC (*p* = 0.003). The worst skin fibrosis, trismus and ear toxicities were similar between the two groups who received IMRT with or without CC. There were 18 patients (7.3%) developing MRI-diagnosed late radiation brain injury; of them, 16 patients had temporal lobe damage injury; two patients had brain stem damage.

**Table 3:** Toxic effects in nasopharyngeal carcinoma patients underwent IMRT with or without concurrent chemotherapy

Subgroup	Toxic effects, No. (%)				
	Without CC(264)		With CC (264)		*P* value
	1-2	3-4	1-2	3-4	
Acute toxic effects during IMRT	264		264		
Leukopenia	150(56.8)	18(6.8)	136(51.5)	74(28.0)	<<0.001
Thrombocytopenia	79(29.9)	0	102(38.6)	16(6.1)	<<0.001
Mucositis	179(67.8)	74 (28)	154(57.5)	103(39.0)	0.023
Nausea/vomiting	42(15.9)	8(3.0)	108(40.9)	40(15.2)	<<0.001
Late toxic effects	116		128		
Ear(deafness/otisis)	62(53.4)	6(5.2)	80 (62.5)	8(6.3)	0.258
Skin fibrosis	72(62.1)	3(2.6)	78(60.9)	3(2.3)	0.994
Trismus	7(6.0)	0	9(7.0)	0	0.801
Salivary glands	48(41.2)	2(1.7)	78(60.9)	4(3.1)	0.004
Brain	6(5.2)	1(0.8)	8(6.3)	3(2.3)	0.612

**Figure 3:**
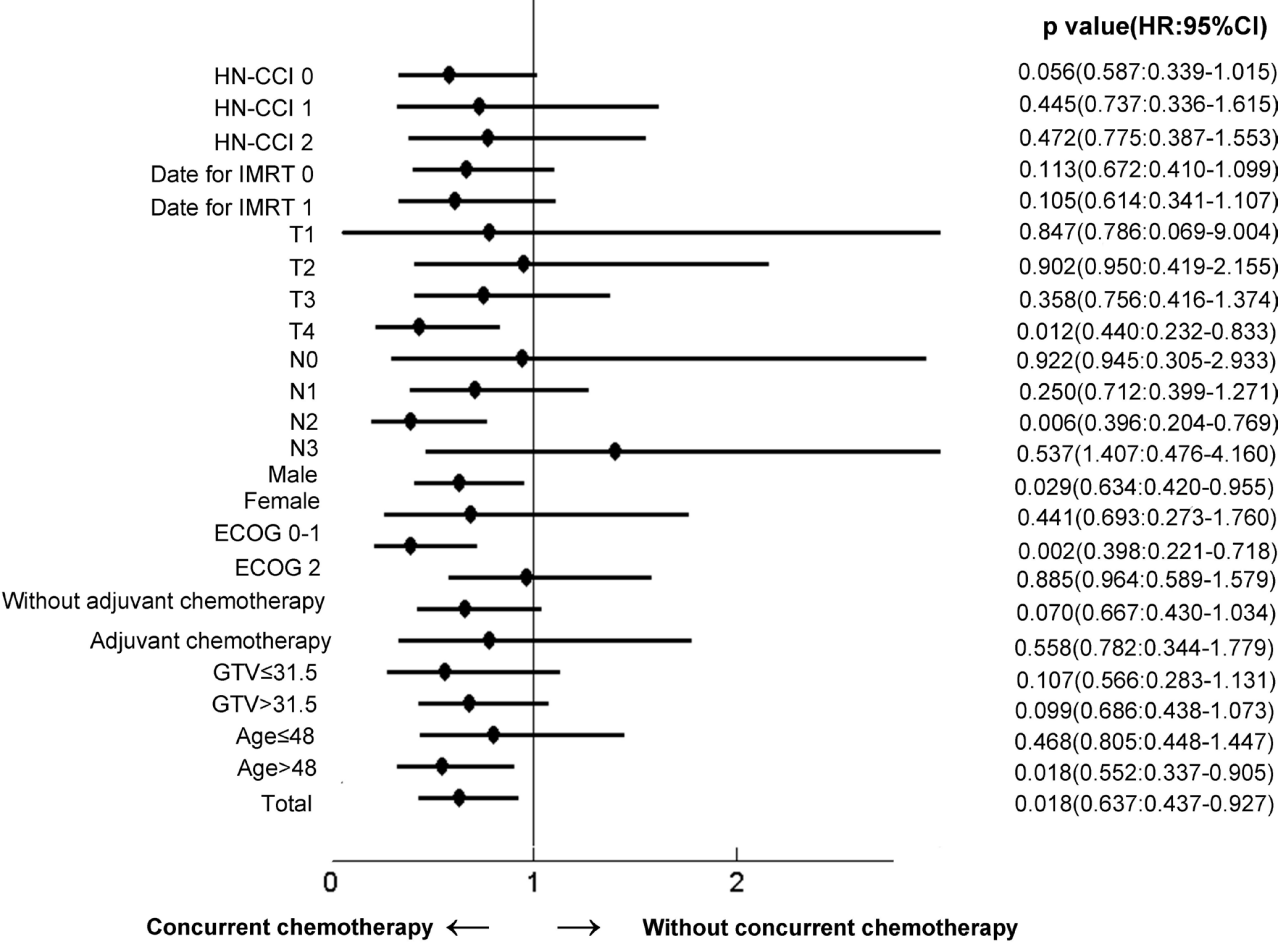
Effects of subgroups on overall survival in nasopharyngeal carcinoma underwent IMRT with versus without concurrent chemotherapy

## DISCUSSION

In previous studies used conventional two-dimensional radiotherapy, significant and clinically relevant improvements with the addition of CC had been confirmed in the treatment of NPC [13,14]. However, with IMRT, the long-term efficacy and safety of CC remains controversial. In a recently published MAC-NPC meta-analysis using individual patient data, the improvement of clinical outcome with CC were verified once again [15]. However, only one-fourth of the patients in the study were administrated with IMRT, and the applicability of the benefits of CC was not clear. Up to now, there is no randomized study to compare the survival outcomes and toxicities of IMRT with and without CC in patients with NPC.

In this study, the benefit of CC was also observed based on IMRT techniques, but the potential beneficial patients were changed to those with T4 or N2 classification. In the era of two-dimensional radiotherapy, the benefits of CC mainly existed in the low-risk patients with T3-4N0-1 disease in some subgroup analysis [16-18]. The disappeared predictive value of T3N0-1 disease might be attributed to delivery of high doses to gross tumor and subclinical disease precisely, due to the advance of contemporary imaging and radiation techniques. However, T4 disease has more biologically aggressive behavior that might relate to stem cell characteristics and radiation resistance, which could not be eliminated by improved radiation dose and coverage. For those NPC patients treated with IMRT, the integration of CC is conducive to tumor control through a synergistic effect that different from the synchronous sensitization mechanism possibly. Compared with the findings in the era of two-dimensional radiotherapy, the emerging benefits of CC in the patients with N2 disease might be attributed to the improvement of radiotherapy and the application of adjuvant chemotherapy (73% of the patients in this study). With regard to the high-risk groups of N3 disease, the difference was not significant, indicating that CC was inadequate and more aggressive combined regimens might be needed.

IMRT has been shown to be superior to conventional radiotherapy in minimizing xerostomia and maintaining quality of life in many trials. However, considerable acute toxicities were accompanied when CC was delivered with IMRT, as observed in this study. In a recently reported study of 868 NPC patients treated with IMRT, significantly severer mucositis, xerostomia and tympanitis were experienced in those patients administrated with CC [7]. Furthermore, the high incidence rate of severe acute toxicity related to concurrent chemoradiotherapy potentially influenced subsequent adjuvant chemotherapy, leading to the difficult in complying fully to planned chemotherapy dosage. In a phase III trial aimed to evaluate the effect of adjuvant chemotherapy in the CC setting, only 63% of patients completed the planned chemotherapy, which can be used partially to explain the lack of benefits from adjuvant chemotherapy in that study [19]. Therefore, a better patient stratification for the administration of CC is clearly needed in the future studies.

Currently, distant metastasis is predominate pattern of failures in the treatment of NPC [4,5,7,20,21]. In this context, the use of systemic treatment to reduce the occurrence of metastatic relapse became more and more important, which highlight the importance of improvising patient stratification to minimize CC related toxicity and optimize the schedule of IMRT and systemic chemotherapy. Besides TNM staging system, a potential strategy is the incorporation of prognostic biomarkers of selecting patients at risk for locoregional relapse, which is warranted in the future studies.

Due to the nature of the study, limitations are inherent to retrospective studies, such as unknown selection bias and inconsistent interval of follow-up. It was the reason why we used propensity score matching to balance the baseline characteristics, as possible as to reduce the potential confounding factors. Also because of this, we lost the opportunity to observe the effects of CC on the compliance of adjuvant chemotherapy. In this study, we also found that patients with good performance status, male and age > 48 years significantly benefit from CC, which might be related to the good tolerance to aggressive therapy in those patients and some unknown biological factors in epidemic areas. However, given the nature of the study, the results should be interpreted cautiously, and need to be confirmed in prospective trials.

## CONCLUSIONS

Our study found that the addition of CC significantly improved the treatment outcomes of NPC patients treated with IMRT, and the benefit was more likely to be observed in patients with T4 and N2 classification. Additionally, acute and late toxicities were increased obviously when CC was administrated with IMRT. These findings indicated that tailored CC and optimizing schedule of IMRT and systemic therapy were needed in future studies, provided that distant metastasis was the predominant pattern of failure in patients treated with IMRT.
